# How Children with Kawasaki Disease Take Acetylsalicylic Acid Mini-Tablets at Home for the Prescribed Period

**DOI:** 10.3390/jcm15010157

**Published:** 2025-12-25

**Authors:** Fuka Serizawa, Iori Taki, Taigi Yamazaki, Nao Tagawa, Chie Arai, Yuki Okada, Taro Kamiya, Takehiko Sambe, Akihiro Nakamura, Tsutomu Harada, Noriko Hida

**Affiliations:** 1 Department of Clinical Research and Development, Graduate School of Pharmacy, Showa Medical University, Tokyo 157-8577, Japant.yamazaki@cmed.showa-u.ac.jp (T.Y.); 2 Department of Hospital Pharmaceutics, Graduate School of Pharmacy, Showa Medical University, Tokyo 142-8666, Japan; 3 Department of Pharmacy, Showa Medical University Hospital, Tokyo 142-8666, Japan; 4 Department of Pediatrics, Graduate School of Medicine, Showa Medical University School of Medicine, Tokyo 142-8666, Japan; 5 Research Administration Center, Showa Medical University, Tokyo 142-8555, Japan; 6 Hatanodai Campus, Showa Medical University, Tokyo 142-8555, Japan; hironak@pharm.showa-u.ac.jp; 7 Clinical Research Institute for Clinical Pharmacology and Therapeutics, Showa Medical University, Tokyo 157-8577, Japan; tharada@pharm.showa-u.ac.jp

**Keywords:** acetylsalicylic acid, mini-tablets, swallowing-related events, mood changes, pediatric dosage form, medication administration completeness, administration pattern, actual medication administration status, Kawasaki disease

## Abstract

**Background/Objectives**: Mini-tablets have gained popularity as a pediatric dosage form owing to their high acceptability. Since 2022, the Showa University Hospital has prescribed acetylsalicylic acid (ASA) mini-tablets to pediatric patients with Kawasaki disease (KD). In this study, we investigated the real-world, at-home administration status of ASA mini-tablets in pediatric patients with KD. **Methods**: This retrospective study included 14 pediatric patients with KD on ASA mini-tablet therapy between November 2022 and October 2024. Medication administration completeness, mood changes during administration, administration patterns, beverages consumed, and swallowing-related events were analyzed. Associations between changes in the administration pattern or beverage consumption and swallowing events or mood changes were evaluated. Serious adverse events and coronary artery aneurysms were assessed using medical records. **Results**: Patients were prescribed ASA mini-tablets for a mean duration of 60.9 days. No serious adverse events or coronary aneurysms were observed. Among the 679 medication records, 5 swallowing-related events were identified. No mood changes following administration were observed in >90% of cases. The mood worsened to “Bad” once, with no further deterioration. The “All at once” administration pattern occurred in 64% of occasions across 12 patients (age: 9–79 months). Patients aged <3 years used medication-assisted jelly, whereas older patients mostly used water. **Conclusions**: ASA mini-tablets can be safely administered at home with minimal swallowing problems. Patients completed full doses irrespective of tablet number, age, administration pattern, or beverage, supporting ASA mini-tablets as an acceptable dosage form option for ASA in KD.

## 1. Introduction

In Japan, powder formulations are primarily prescribed dosage forms for children aged <6 years [1]. However, a Japanese and Dutch study reported that administering powder formulations can be challenging in some children [2,3]. Similarly, a Dutch study found that children aged 1–4 years considered powder formulations less acceptable than other dosage forms. To address these issues, mini-tablets with diameters of 2–4 mm have recently gained attention as novel pediatric dosage forms. Mini-tablets are recognized as a pediatric dosage form according to the Guideline on pharmaceutical development of medicines for pediatric use issued by the European Medicines Agency in 2013 [4]. Pediatric formulations should be easy to administer and palatable [5]. Mini-tablets reportedly have higher acceptability than other pediatric dosage forms. A European study reported that children aged 1 month to 12 years showed higher acceptability for placebo mini-tablets than for syrup [6,7]. In a trial conducted in Japan involving children aged 6–23 months, even those aged 6–11 months showed higher acceptability for placebo mini-tablets than for fine granules and syrup [8]. Conversely, one study reported that Japanese children aged 2–3 years preferred syrup over placebo mini-tablets [9]. Since 2024, Uptravi^®^ Tablets (0.05 mg, diameter 3.1 mm) and Entresto^®^ Granular Tablets (12.5 mg/31.25 mg, diameter 2 mm) have been launched for pediatric use in Japan [10,11]. In Europe, Slenyto^®^ Prolonged-Release Tablets (1 mg/5 mg, diameter 3 mm) and Aqumeldi^®^ Dispersible Tablets (0.25 mg/1 mg, diameter 2 mm) were launched in 2018 and 2023, respectively [12,13]. Given that mini-tablets are now used in clinical practice, the focus has shifted from evaluating their acceptability to examining actual medication administration in daily life settings.

Kawasaki disease (KD) is a pediatric vasculitis that mainly affects boys aged 0–4 years in Asia [14,15,16]. To prevent coronary artery lesions (CALs), a serious KD complication, ASA is usually administered for 2–3 months following the acute phase [17,18]. In Japan, the only pediatric dosage form of acetylsalicylic acid (ASA), which has a sour taste, is the powder formulation [19], which may reduce medication adherence and consequently affect long-term continuous administration. Since 2022, Showa University Hospital has compounded and prescribed at-home ASA mini-tablets for pediatric patients with KD to improve medication adherence [20]. To monitor medication status post-discharge, caregivers are requested to maintain a medication logbook [20].

We recently conducted a pilot study involving three pediatric patients with KD, which demonstrated the feasibility of the long-term administration of ASA mini-tablets with high medication adherence [20]. Previous studies have evaluated pediatric formulation preferences based on mood after medication as a metric [6,7]. In contrast, this study focused on mood before and after medication to better capture mood changes and confirm the acceptability of mini-tablets. In Japan, nurses and parents use various strategies when administering medications to children, such as using syrups, administration aids, or combining doses into a single administration [21]. Accordingly, this study also examined administration patterns and beverages consumed during ASA mini-tablet administration at home, and particular attention was given to age-related differences in swallowing ability [22]. This study aimed to clarify the real-world at-home administration status of ASA mini-tablets in pediatric KD patients, focusing on completeness of medication administration, mood before and after ASA mini-tablet administration and its changes, administration patterns, beverages used, and swallowing-related events during ASA mini-tablet administration.

## 2. Materials and Methods

### 2.1. Research Design and Ethical Considerations

This retrospective observational study included pediatric patients diagnosed with KD between November 2022 and October 2024. This study was conducted in accordance with the Declaration of Helsinki and approved by Showa University Research Ethics Review Board (Approval No. 2024-051-A, 17 May 2024). As this study utilized existing data, participants were publicly notified of an opt-out consent process, providing them the option to decline participation.

Approval for the therapeutic use of ASA mini-tablets was obtained from Showa University Hospital Evaluation Committee for High-Risk New Medical Technologies and Unapproved New Medical Practices (Approval No. 2205-P-00005, 19 July 2022). Informed consent for treatment was obtained from the guardians.

### 2.2. Selection of Pediatric Patients

The following selection criteria were implemented:

1. Pediatric patients diagnosed with KD at the Department of Pediatrics or Pediatric Cardiology, Showa University Hospital, between November 2022 and October 2024;
2. Patients who could take placebo mini-tablets;
3. Patients who received ASA mini-tablet treatment;
4. Patients for whom a medication logbook maintained by caregivers post-discharge was available.

Pediatric patients enrolled in other clinical studies were excluded from this study.

### 2.3. ASA Mini-Tablets and KD Treatment

#### 2.3.1. Compounding and Controlling the Quality of ASA Mini-Tablets

The 3 mm diameter ASA mini-tablets used in this study were compounded at the Showa University Hospital following the method described in our previous study [20]. These mini-tablets were prescribed to patients diagnosed with KD.

Each ASA mini-tablet was composed of ASA 10 mg (Yoshida Pharmaceutical Co., Ltd., Tokyo, Japan); crystalline cellulose 2 mg (Theolas™ KG1000; Asahi Kasei Corporation, Tokyo, Japan); corn starch 2 mg (Yoshida Pharmaceutical Co., Ltd., Tokyo, Japan); D-mannitol 5.4 mg (Parteck^®^ M200; Merck KGaA, Darmstadt, Germany); and magnesium stearate 0.6 mg (Taihei Chemical Industry Co., Ltd., Osaka, Japan). The mini-tablets were manufactured using a single-stroke tablet press (N-30E; Okada Seiko Co., Ltd., Osaka, Japan).

Comprehensive quality control tests were conducted to confirm compliance with the Japanese Pharmacopoeia standard [23]. These included disintegration, content uniformity, dissolution, hardness, and weight uniformity tests [20]. Furthermore, the ASA mini-tablets used in this study were confirmed to exhibit no significant differences in plasma drug concentrations compared with ASA powder formulations in adults [24].

#### 2.3.2. Treatment of KD with ASA Mini-Tablets and Medication Guidance

During the acute phase of KD, pediatric patients received ASA powder or mini-tablets at 30 mg/kg/day, divided into three daily administrations, in accordance with established Guidelines for Medical Treatment of Acute KD and ASA package insert [17,19]. This treatment was combined with intravenous immunoglobulin (IVIG) and other medications based on the treatment resistance prediction score [17,18]. After 48 h of fever resolution, the dose was reduced to 5 mg/kg/day once daily for a period of 2–3 months [18,19]. Prior to initiating ASA mini-tablet administration, each patient’s ability to ingest placebo mini-tablets was confirmed during hospitalization [20].

During hospitalization, the parents received instructions from nurses on the handling and administration of ASA mini-tablets. Given that the ASA package insert does not specify detailed administration instructions [19], the administration pattern and choice of beverage were left to the discretion of the caregivers. Upon discharge, pharmacists provided guidance to parents on the number of tablets, storage conditions, and appropriate actions to be taken in case of fever.

### 2.4. Data Collection

#### 2.4.1. Medication Logbook

Caregivers were instructed to begin recording entries in the medication logbook from the initiation of ASA mini-tablet administration. For this study, post-discharge logbook records were collected to assess actual at-home medication administration of the pediatric patients.

The logbooks were managed by the caregivers, who were instructed to document each dose immediately after the administration, and were recorded in chronological order, although no formal training on recording procedures was provided. The record period spanned from the initiation of ASA mini-tablet administration post-discharge until discontinuation as directed by the attending physician. The six items recorded in the medication logbook are detailed in Table 1. In addition, caregivers could report any concerns or observations in the free-form comment section.

#### 2.4.2. Medical Records

Medical records were collected from KD diagnosis through 3 months post-onset. The records included the following:

- Sex;
- Age;
- Height;
- Weight;
- Body temperature;
- Duration and dosage of ASA powder and mini-tablet prescriptions;
- Concomitant medications;
- IVIG treatment resistance prediction score (Kobayashi score);
- Severity assessment;
- Blood tests (serum Na, C-reactive protein, white blood cell count, platelet count, aspartate aminotransferase level, and neutrophil count);
- Coronary artery diameters (right coronary artery [RCA], left main trunk [LMT], left anterior descending artery [LAD], and left circumflex artery [LCX]).

### 2.5. Actual Medication Administration Status of ASA Mini-Tablets

#### 2.5.1. Adverse Events, ASA Mini-Tablets Prescription Duration, and Medication Logbook Recording Rate

Adverse events associated with ASA mini-tablets were evaluated using the medical records of the pediatric patients included in this study. The average number of days for which ASA mini-tablets were prescribed was calculated. Subsequently, assessment of the medication logbook focused on the percentage of logbook entries recorded by caregivers relative to the prescribed duration for each patient, with the mean recording rate documented for the cohort.

#### 2.5.2. Swallowing-Related Events and Medication Administration Completeness During ASA Mini-Tablet Administration

Swallowing-related events were defined from the medication logbook entries during administration, with events categorized as “Chewed,” “Coughed,” “Refused,” or any otherwise noted in the free-form comment section. Medication administration completeness was determined based on whether the full prescribed single dose of ASA mini-tablets was utilized. In case of inconsistencies in the records, entries were revised for accuracy; for example, “No problem” accompanied by a comment stating, “Chewed the tablet before swallowing,” was recategorized as “Chewed.”

#### 2.5.3. Mood Before and After ASA Mini-Tablet Administration and Its Changes

Mood changes were evaluated by comparing the mood entries before and after ASA mini-tablet administration. Transitions were descriptively classified as: “Improved mood” (post ≥ pre; e.g., Usual → Good, Bad → Good/Usual), “No change in mood” (post = pre), or “Worsened mood” (post < pre; e.g., Good → Usual/Bad). For example, when the pre-administration mood was “Usual” and the post-administration mood was “Good,” this was interpreted as “improved mood.” Note that “Usual” lacks a defined ordinal position relative to “Good”/“Bad,” limiting interpretations to descriptive transitions rather than definitive clinical improvement/deterioration.

#### 2.5.4. Administration Patterns and Beverages Used During ASA Mini-Tablet Administration

We examined the administration pattern for ASA mini-tablets (classified as “One tablet at a time,” “In several doses,” or “All at once”) and the type of beverage used during ASA mini-tablet administration (categorized as “Water,” “Medication-assisted jelly,” or “Other beverages”). We investigated the occurrence of swallowing-related events and mood changes in case of discrepancies in administration pattern or beverage use compared with previous administration, using logbooks that were recorded in chronological order.

#### 2.5.5. KD Severity Assessment

The severity of coronary artery disease was assessed using the Z-score, as defined by the American Heart Association in 2017 guidelines [18]. Sex, height, weight, and coronary artery diameter measurements were converted to Z-scores using LMS 4 (Z-Score Project) version 4.0 software [25]. A lesion with a Z-score of +2.5 or higher was defined as significant [18].

### 2.6. Statistical Analysis

Descriptive statistics, including means and standard deviations, were calculated using Microsoft Excel (Microsoft Corporation, Redmond, WA, USA). Incomplete entries were deemed “incomplete entries” and excluded from the analysis.

## 3. Results

### 3.1. Patient Background

Medication logbooks were collected from 14 pediatric patients with KD, comprising 9 boys and 5 girls, who were prescribed ASA mini-tablets. The characteristics of these patients are detailed in Table 2. The mean age at admission was 44.6 months, with a range of 9–83 months. The mean number of ASA mini-tablets taken per dose was 7.3, with a range of 5–10 tablets. During hospitalization, 13 of the 14 patients received maintenance-dose ASA mini-tablets. The mean Kobayashi score, which predicts resistance to IVIG therapy, was 3.4, ranging from 0 to 10 points [18]. Three patients who were resistant to IVIG therapy were additionally treated with IVIG, cyclosporine, and ASA at the time of admission. All participants were experiencing their first episode of KD. Data on body temperature and laboratory test results at admission are provided in Appendix A Table A1.

### 3.2. Adverse Events, ASA Mini-Tablets Prescription Duration, and Medication Logbook Recording Rate

The mean post-discharge prescription duration for ASA mini-tablets was 60.9 days, with a range of 51–72 days. All pediatric patients were prescribed ASA mini-tablets for the therapeutically necessary duration post-discharge and completed their KD treatment. No serious adverse events or KD recurrence were observed during the ASA mini-tablet administration period. Furthermore, none of the caregivers requested a change in the dosage form. The mean medication logbook recording rate among patients was 78.7%, with a range of 30–100%. A total of 686 medication records, including incomplete entries, were collected from all patients.

### 3.3. Swallowing-Related Events and Medication Administration Completeness During ASA Mini-Tablet Administration

A summary of swallowing-related events and medication administration completeness among 679 medication records is provided in Table 3. Medication administration completeness was classified as “All ASA mini-tablets” or “Partial dose.” Of the 679 records, 674 had no swallowing-related events, of which 670 documented successful administration of all prescribed ASA mini-tablets.

Swallowing-related events recorded included “Chewed” in one patient on Day 20, “Coughed” in one patient on Days 5 and 8, “Spat out” in one patient on Day 14, and “Refused” in one patient on Day 21. All pediatric patients who experienced swallowing-related events continued their ASA mini-tablet regimen during subsequent visits.

In addition, there were four instances in which a partial dose was taken and no swallowing-related events occurred: one due to tablet damage, and three due to unknown reasons. Seven medication logbook entries were incomplete regarding swallowing-related events and the medication administration completeness.

### 3.4. Mood Before and After ASA Mini-Tablet Administration and Its Changes

A summary of mood assessments before and after ASA mini-tablet administration across 674 medication records is provided in Table 4. Mood changes were classified as “Improved mood,” “No change in mood,” or “Worsened mood.” “No change in mood” was recorded in 619 instances (91.8%), with 448 “Good to Good,” 170 “Usual to Usual,” and 1 “Bad to Bad.” “Improved mood” was recorded in 25 instances (3.7%), with 8 “Usual to Good,” 5 “Bad to Good,” and 12 “Bad to Usual.” “Worsened mood” was recorded in 30 instances (4.5%), with 29 “Good to Usual” and 1 “Usual to Bad.” Twelve medication logbook entries were incomplete regarding mood changes. In the two cases where mood worsened to “Bad” post-administration, neither patient exhibited a “Bad” mood on the following day or thereafter.

Mood changes recorded on the first day post-discharge accounted for 14 instances: 12 instances of “No change in mood,” 1 “Improved mood,” and 1 “Worsened mood.” These included 9 “Good to Good,” 3 “Usual to Usual,” 1 “Usual to Good,” and 1 “Good to Usual” transitions.

### 3.5. Administration Patterns and Beverages Used During ASA Mini-Tablet Administration

The overall distribution of administration patterns was analyzed across 681 medication records, and a detailed summary by age group for each pediatric patient is provided in Table 5. Administration patterns were recorded as 70 instances of “One tablet at a time” (10.3%), 173 instances of “In several doses” (25.4%), and 438 instances of “All at once” (64.3%). Five medication logbook entries were incomplete regarding the administration pattern. Among the 14 pediatric patients, five used “One tablet at a time” at least once, ranging from 14 months (57/58 doses, 5 tablets per dose) to 79 months (1/36 doses, 10 tablets per dose). No patient consistently used this pattern throughout the study. Eight patients used the “in several doses” at least once, ranging from 9 months (6/59 doses, 5 tablets per dose) to 83 months (53/53 doses, 10 tablets per dose), with only one patient using it consistently. Twelve patients used the “All at once” at least once, ranging from 9 months (53/59 doses, 5 tablets per dose) to 79 months (33/36 doses, 10 tablets per dose), and four patients used it consistently. Swallowing-related events associated with administration patterns included one instance of “Spat out” for “All at once,” two instances of “Coughed” for “In several doses,” and one instance each of “Chewed” and “Refused” for “One tablet at a time.”

The overall distribution of beverage used during ASA mini-tablet administration was analyzed across 656 medication records, and a detailed summary by age group for each pediatric patient is provided in Table 5. Beverages used were recorded as 292 instances of “Water” (44.5%), 174 instances of “Medication-Assisted jelly” (26.5%), and 190 instances of “Other beverages” (29.0%). The “Other beverages” category included tea, milk, syrup, yogurt, and fruit juice. Thirty medication logbook entries were incomplete regarding the beverage used. Among the 656 records, water was predominantly used by patients aged ≥24 months, with 233 of 304 instances (76.6%) recorded in those aged ≥36 months. Medication-assisted jelly was mainly used by patients aged 12–36 months, accounting for 174 of 293 instances (59.4%). No clear age-related trends were observed in the use of other beverages.

Among all medication records, changes in administration patterns from the previous instance were recorded in 47 instances. Of these, three cases corresponded with mood changes differing from the prior instance, with each instance recorded in a different patient. In one case, mood remained stable as “Good to Good” (no change) with “In several doses” but improved from “Bad to Usual” upon switching to “One tablet at a time.” In another, mood worsened from “Good to Usual” with “In several doses” but improved from “Bad to Usual” when switching to “All at once.” In the third case, mood was stable as “Good to Good” (No change in mood) with “All at once” but worsened to “Good to Usual” upon switching to “In several doses.” No swallowing-related events were reported during these administration pattern changes.

Changes in beverages from the previous instance were recorded in 48 instances. Two of these involved mood changes that were recorded in the same patient. In one instance, mood remained stable as “Good to Good” (no change) with yogurt but improved from “Bad to Usual” upon switching to fruit juice. During this mood improvement, the patient refused the full dose, consuming only part of it. The subsequent return to yogurt as the beverage was associated with mood stabilization (“Good to Good”), and no further swallowing-related events were reported during this period.

### 3.6. KD Severity Assessment

At initial hospital admission, Z-scores for all pediatric patients remained below +2.5, with no significant coronary artery dilatation or CAL. At 3 months post-onset, coronary artery diameters were as follows: RCA, 2.0 ± 0.4 mm (1.4 to 2.6 mm); LMT, 2.2 ± 0.4 mm (1.5 to 2.8 mm); LAD, 1.9 ± 0.4 mm (1.1 to 2.6 mm); LCX, 1.7 ± 0.2 mm (1.3 to 2.1 mm). The corresponding Z-scores were as follows: RCA, 0.4 ± 0.7 (−0.8 to +1.5); LMT, 0.2 ± 0.8 (−1.3 to +1.5); LAD, 0.4 ± 0.9 (−1.7 to +1.5); LCX, 0.4 ± 0.5 (−0.5 to +1.0). No cases of CAL were observed in any coronary arteries, resulting in a 0% CAL incidence rate. Furthermore, cardiac ultrasound examinations conducted 1 year post-onset in 8 of the 14 participants showed no evidence of CAL.

## 4. Discussion

To the best of our knowledge, this study is the first to clarify the actual at-home administration status of ASA mini-tablets in pediatric patients with KD. To elucidate this, we examined medication administration completeness, mood before and after administration and its changes, administration patterns, beverages used, and swallowing-related events during ASA mini-tablet administration. The key finding was that patients were able to take their prescribed ASA mini-tablets at home without worsening mood or swallowing-related events, suggesting that appropriate caregivers’ support was provided throughout the observation period.

We focused on the presence or absence of swallowing-related events and medication administration completeness because adherence to the prescribed ASA dose is critical for KD treatment. Swallowing-related difficulties can undermine continued treatment. Our previous study reported that 33% of children aged 2–3 years swallowed mini-tablets without chewing [9], whereas in this study, 98% of medication instances among patients aged 9–83 months were free of swallowing-related events, and all patients had completed the medication course. Thirteen of fourteen patients were prescribed ASA mini-tablets after confirming their ability to take placebo mini-tablets during hospitalization, supporting that clinical experience facilitates safe home administration.

Mood data before and after administration showed high acceptability. Among 674 entries, pre-administration moods were “Good” in 70.8%, “Usual” in 26.5%, and “Bad” in 2.7%. Post-administration moods were “Good” in 68.3%, “Usual” in 31.3%, and “Bad” in 0.3%. Notably, all “Bad” pre-administration moods improved post-dose. Mood changes comprised “Improved mood” in 3.7% and “No change” in 91.8% of instances. These results align with our prior pilot study [20] and extend previous post-administration only evaluations through bi-temporal measurement [6,7].

Regarding administration patterns, 64% of instances involved taking tablets “All at once,” and 36% involved dividing the dose. Despite varied administration methods [21], all participants successfully ingested the full dose. Even the youngest patient, aged 9 months, tolerated a full single administration. These findings demonstrate that ASA mini-tablets are suitable for administering complete doses across a wide pediatric age range. Water was the preferred beverage for patients aged ≥48 months, whereas medication-assisted jelly was more common in those <36 months, which is reflective of the typical Japanese pediatric medication practices [21]. Caregivers likely selected beverages based on patients’ swallowing abilities and preferences, facilitating successful adherence.

No participant developed CAL within 3 months post-KD onset. No adverse events or KD recurrence occurred during ASA administration. Given ASA’s reduction to maintenance dose post-fever resolution [18,19], early hospital assessment supports the feasibility of ASA mini-tablets for ongoing KD treatment. However, fine-tuning ASA dosage with mini-tablets is difficult because each tablet contains 10 mg. Some studies indicate that ASA dosage does not increase the risk of CAL [17,26], suggesting that precise dosage adjustment may not be the highest priority. Thus, ASA mini-tablets represent a highly acceptable formulation in the maintenance phase.

This study builds on our previous study demonstrating ASA mini-tablets’ long-term feasibility [20] and adherence by providing detailed home administration data, including medication administration completeness, mood changes, administration patterns, beverages used, and swallowing-related events during administration. Study limitations include retrospective design and possible selection bias, with incomplete detailed records in case of swallowing-related or mood events, limiting the robustness of data. The medication logbooks were recorded subjectively by caregivers, and therefore the data are not fully objective. Key limitation is the subjective 3-category mood scale (“Good/Usual/Bad”), where “Usual” lacks a defined ordinal position. In particular, mood before and after ASA mini-tablet administration and its changes may not be reliably assessed. The single-center and small sample size (*n* = 14) limit the generalizability of its results to broader KD pediatric populations.

Future prospective studies are needed to provide evidence on whether ASA mini-tablets enhance adherence across large pediatric populations. This is a necessary consideration for the widespread adoption of mini-tablets as one of the dosage form options for the maintenance dose of ASA in KD. In addition, comparative studies between at-home ASA powder and mini-tablets are warranted to delineate differences in medication administration status.

## 5. Conclusions

This study provides evidence that ASA mini-tablets were safely and consistently accepted by pediatric patients for home use, without causing mood changes. The results highlight the potential of mini-tablet formulations as a feasible dosage form for the maintenance dose of ASA in KD for routine clinical practice. To further support their widespread adoption, future studies involving larger cohorts and detailed medication records are necessary to generate robust scientific evidence for their superior medication adherence and acceptability. These findings may serve as a foundation for the rational selection of new ASA dosage forms.

## Figures and Tables

**Table 1:** Medication logbook items recorded for pediatric patients with Kawasaki Disease receiving acetylsalicylic acid mini-tablets.

Medication Logbook Items	Day___
Mood before ASA mini-tablet administration	□ Good □ Usual □ Bad
Medication administration completeness	□ Completely taken □ ___tablets taken
Events occurring during ASA mini-tablet administration	□ No problem □ Chewed □ Coughed □ Refused
Intake pattern	□ One tablet at a time □ In several doses □ All at once
Beverage used during ASA mini-tablet administration	□ Water □ Medication-assisted jelly □ Other beverages (___)
Mood after ASA mini-tablet administration	□ Good □ Usual □ Bad
Please note any other concerns you may have.	

The medication logbook provided to caregivers included the instruction: “Record information about the mini-tablets and report it to your doctor.” Abbreviations: acetylsalicylic acid (ASA).

**Table 2:** Characteristics of pediatric patients with Kawasaki Disease treated with acetylsalicylic acid mini-tablets (*n* = 14).

Variables	Total (*n* = 14)
**Background characteristics at diagnosis**	
Age (months)	44.6 ± 22.9 (9.0–83.0)
Weight (kg)	14.3 ± 3.4 (9.1–20.0)
Body Mass Index (kg/m^2^)	15.4 ± 1.1 (14.1–18.2)
Sex (male/female)	9/5
**Clinical characteristics**	
Number of ASA mini-tablets administered per dose (tablets)	7.3 ± 1.7 (5–10)
Kobayashi score (points)	3.4 ± 3.0 (0–10)
IVIG-resistant patient	3
**Initial** **coronary artery diameters (mm)**	
RCA (#1)	2.0 ± 0.2 (1.6–2.4)
LMT (#5)	2.2 ± 0.3 (1.7–2.6)
LAD (#6)	1.8 ± 0.4 (1.2–2.4)
LCX (#11)	1.8 ± 0.3 (1.4–2.4)
**Initial Z** **-** **score of coronary arteries**	
RCA (#1)	0.6 ± 0.5 (−0.2–+1.4)
LMT (#5)	0.2 ± 0.8 (−1.8–+1.6)
LAD (#6)	0.3 ± 1.0 (−1.6–+1.8)
LCX (#11)	0.9 ± 0.7 (−0.4–+2.0)

The following are presented as mean ± standard deviation (minimum value–maximum value): age, weight, Body Mass Index, number of ASA mini-tablets taken per dose, Kobayashi score, Z-scores for each coronary artery, and each coronary artery diameter. Abbreviations: acetylsalicylic acid (ASA), intravenous immunoglobulin (IVIG), right coronary artery (RCA), left main coronary artery (LMT), left anterior descending branch (LAD), left circumflex branch (LCX). Note: Vessel numbers (#1, #5, #6, #11) refer to standard coronary artery segment classifications per the American Heart Association guidelines.

**Table 3:** Swallowing-related events by Medication Administration Completeness in pediatric patients with Kawasaki Disease (*n* = 14).

Medication Administration Completeness	No Event Occurred	Event Occurred				Total
		Chewed	Coughed	Spat Out	Refused	
**All ASA mini** **-** **tablets**	670	1	2	1 *	0	674
**Partial dose**	4	0	0	0	1	5
**Total**	674	1	2	1	1	679

* “Spat out” event recorded from free-text logbook entries. Vertical: completeness categories (“All ASA mini-tablets” or “Partial dose”). Horizontal: swallowing-related events (“No event” or specific events). Swallowing-related events under Events under “Occurred” are mutually exclusive per administration. Abbreviations: acetylsalicylic acid (ASA).

**Table 4:** Summary of mood changes before and after acetylsalicylic acid mini-tablet administration in pediatric patients with Kawasaki Disease (*n* = 14).

Before ASA Mini-Tablet Administration	After ASA Mini-Tablet Administration			Total
	Good	Usual	Bad	
**Good**	448	29	0	477
**Usual**	8	170	1	179
**Bad**	5	12	1	18
**Total**	461	211	2	674

The vertical axis shows the mood before ASA mini-tablet administration, and the horizontal axis shows the mood after administration. Abbreviations: acetylsalicylic acid (ASA).

**Table 5:** Age-stratified summary of administration patterns and beverages used during acetylsalicylic acid mini-tablet administration in pediatric patients with Kawasaki Disease (*n* = 14).

	9–11 Months (*n* = 1)	12–23 Months (*n* = 2)		24–35 Months (*n* = 3)			36–47 Months (*n* = 1)	48–59 Months (*n* = 3)			60–71 Months (*n* = 2)		72–83 Months (*n* = 2)		Total
**Administration patterns**															
One tablet at a time	0	57	0	6	0	0	0	5	0	1	0	0	1	0	70
In several doses	6	0	8	54	0	28	0	4	18	0	0	0	2	53	173
All at once	53	1	52	0	63	19	18	11	42	71 *	18	57	33	0	438
**Beverages**															
Water	0	0	0	59	0	0	18	20	34	1	18	57	32	53	292
Medication- Assisted jelly	0	0	60	0	63	51 **	0	0	0	0	0	0	0	0	174
Vegetable juice	0	0	0	1	0	0	0	0	1	0	0	0	0	0	2
Tea	0	0	0	0	0	0	0	0	25	40	0	0	0	0	65
Milk	0	0	0	0	0	0	0	0	0	1	0	0	4	0	5
Syrup	59	1	0	0	0	1	0	0	0	0	0	0	0	0	61
Yogurt	0	54	0	0	0	0	0	0	0	0	0	0	0	0	54
Fruits juice	0	3	0	0	0	0	0	0	0	0	0	0	0	0	3

The upper table shows the administration patterns, and the lower table shows the breakdown of beverages consumed during ASA mini-tablet administration. This breakdown is displayed vertically (in columns) for each patient. * Among these, 30 instances had no records of beverages. ** Among these, 5 instances had no records of the administration pattern. Abbreviations: acetylsalicylic acid (ASA).

## Data Availability

The dataset and analysis data supporting the conclusions of this study will be made available upon reasonable request from the corresponding authors.

## Abbreviations

The following abbreviations are used in this manuscript:

KD	Kawasaki disease
CAL	coronary artery lesions
ASA	acetylsalicylic acid
IVIG	intravenous immunoglobulin
RCA	right coronary artery
LMT	left main coronary artery
LAD	left anterior descending artery
LCX	left circumflex artery

## Appendix A

**Table A1:** Characteristics of pediatric patients with Kawasaki Disease at the time of initial treatment (*n* = 14).

Variables	Total (*n* = 14)
**Clinical characteristics**	
Body temperature (°C)	38.5 ± 0.9
Timing of treatment	
≤4 days of fever	3
>4 days of fever	11
**Laboratory values at diagnosis**	
White Blood Cell count (/μL)	14,292 ± 4517
Neutrophil count (/μL)	10,593 ± 3567
≥80%	5
Platelet count (×10^4^/μL)	32.6 ± 5.3
C-Reactive Protein (mg/dL)	8.3 ± 3.2
Aspartate Aminotransferase (U/L)	202 ± 362
Sodium (mEq/L)	134.0 ± 1.7

The following are presented as mean ± standard deviation (minimum value–maximum value): body temperature, white blood cell count, neutrophil count, platelet count, C-reactive protein, aspartate aminotransferase, and serum sodium.
